# Draft Genome Sequence of Pedobacter sp. Strain NL19, a Producer of Potent Antibacterial Compounds

**DOI:** 10.1128/genomeA.00184-15

**Published:** 2015-03-26

**Authors:** Tiago Santos, Andreia Cruz, Tânia Caetano, Cláudia Covas, Sónia Mendo

**Affiliations:** Biology Department & CESAM, University of Aveiro, Aveiro, Portugal

## Abstract

Here, we report the draft genome sequence of Pedobacter sp. strain NL19. The genome has 5.99 Mbp and a G+C content of 39.0%. NL19 was isolated from sludge from an abandoned uranium mine in the north of Portugal, and it produces potent antibacterials against Gram-positive and Gram-negative bacteria.

## GENOME ANNOUNCEMENT

Pedobacter sp. strain NL19 was isolated from a sludge sample collected from an abandoned uranium mine, Quinta do Bispo, in the Viseu District, Portugal. Due to its 16S rRNA gene sequence, this bacterium was identified as belonging to the genus Pedobacter (GenBank accession no. KJ579161).

The genus Pedobacter belongs to the *Sphingobacteriaceae* family, which includes heparinase-producing, obligatory aerobic Gram-negative stain rods (1). Since the proposal of the Pedobacter genus, 46 species have been described in the List of Prokaryotic Names with Standing in Nomenclature (accessed on 2 September 2015) (2).

Pedobacter sp. NL19 exhibits potent antibacterial activity *in vitro* against relevant Gram-positive and Gram-negative bacteria, including Aeromonas hydrophila ATCC 7966, Listeria monocytogenes 71, and Klebsiella pneumoniae ATCC 700603 (an extended-spectrum beta-lactamase producer).

Total DNA was extracted with the DNeasy blood and tissue kit (Qiagen) according to the manufacturer's instructions. The genome was sequenced using the platform Ion PGM system generating 3,722,256 reads (with approximately 128× coverage). Using CLC Genomics Workbench 7.0.3, the high-quality reads sequences were assembled in 201 contigs, ranging from 499 to 180,550 bp in length, with an *N*_50_ of 57,428. The draft genome contains 5,988,703 bp and a G+C content of 39.0%.

Functional annotation of the assembled genome was performed with the RAST 2.0 (Rapid Annotation using Subsystem Technology) service (3), tRNA genes were predicted using tRNAscan-SE-1.23 (4), and rRNA genes were detected by RNAmmer 1.2 server (5). The annotation predicted a total of 5,382 protein encoding genes, 52 tRNA genes, and 5 rRNA genes (three 5S, one 16S, and one 23S). Since this is a draft, the total numbers and locations of multiple copies of RNAs are not correctly identified.

The closest-neighbors tool, available in RAST through functional comparison of the genome, revealed that strain NL19 is more closely related to Pedobacter sp. BAL39 (similarity score value, 541), Pedobacter heparinus DSM 2366 (score value, 538), Sphingobacterium spiritivorum ATCC 33861 (score value, 468), S. spiritivorum ATCC 33300 (score value, 467), and Pedobacter saltans DSM 12145 (score value, 435).

According to the RAST annotation, NL19 possesses genes encoding antibiotic resistance to beta-lactams (15 genes) and fluoroquinolones (4 genes). In addition, genes encoding for multidrug resistance efflux pumps (23 genes) are also present. Genetic determinants associated with resistance to heavy metals (3 arsenic genes, 1 zinc gene, and 40 cobalt-zinc-cadmium genes) are also present. AntiSMASH 3.0 (6) identified 21 clusters for secondary metabolites (including six clusters for lanthipeptides, seven clusters for nonribosomal peptide synthetases [NRPS], two clusters for siderophores, one cluster for polyketide synthetase [PKS], one cluster encoding a terpene, and 1 cluster encoding a linaridin) in the genome of NL19, revealing the potential of this strain to produce a wide range of biotechnological relevant compounds.

### Nucleotide sequence accession numbers.

This whole-genome shotgun project was deposited at DDBJ/EMBL/GenBank under the accession no. JXRA00000000. The first version is described herein, JXRA01000000.
